# Preparation, Characterization and Application of UV-Curable Flexible Hyperbranched Polyurethane Acrylate

**DOI:** 10.3390/polym9110552

**Published:** 2017-10-25

**Authors:** Hongping Xiang, Xiaowei Wang, Guanghong Lin, Lu Xi, Yan Yang, Dehua Lei, Haihui Dong, Jiahui Su, Yanyan Cui, Xiaoxuan Liu

**Affiliations:** Guangdong Provincial Key Laboratory of Functional Soft Condensed Matter, School of Materials and Energy, Guangdong University of Technology, Guangzhou 510006, China; lyzxhp@163.com (H.X.); wxw2423@163.com (X.W.); colin616647399@163.com (G.L.); 13798197446@163.com (L.X.); ykkai123@126.com (Y.Y.); 13822339144@163.com (D.L.); d373670858@163.com (H.D.); sujiahuigdut@outlook.com (J.S.); lunwenpfp@126.com (Y.C.)

**Keywords:** hyperbranched polyurethane acrylate, UV-curing, flexibility, coatings, reinforcements

## Abstract

A novel UV-curable hyperbranched polyurethane acrylate (FHBPUA) with excellent flexibility is successfully synthesized based on a reaction of hydroxyl terminated hyperbranched polyurethane (regarded as core) with flexible semiadduct urethane monoacrylate (regarded as arms). The structure and property of FHBPUA is firstly analyzed and then utilized as functional additives to ameliorate the UV-curing and mechanical properties of epoxy acrylate resin. The degree of branching of FHBPUA turns out to be 0.82. Its thermal decomposition process consists of three different stages, and the glass transition temperature is around 65 °C. The freestanding FHBPUA film (~30 μm thickness) can be UV-cured within 3 s, and its flexibility is up to 1 mm. With the increase of FHBPUA content to 10 wt %, the UV-curing time of UV1000 film decreases from 6 to 3 s, flexibility strikingly increases from 10 to 1 mm, and adhesive force also improves from 5 to 3 grades, meanwhile its glossiness is not influenced by FHBPUA. In addition, a certain amount of FHBPUA can improve the tensile strength and elongation at break of UV1000 film. This novel FHBPUA can be used not only to develop flexible UV-curable freestanding films but also as functional additives to perfect other UV-curable compositions like coatings, inks and 3D printed parts.

## 1. Introduction 

In recent years, more and more attention has been given to hyperbranched polymers (HBPs) due to their highly branched three-dimensional spherical architectures, and unique physical and chemical properties such as plentiful terminal functional groups, low viscosity and high solubility. HBPs can be facilely synthesized by various approaches including “one-pot” polymerization of AB_x_ (x ≥ 2) monomers, self-condensing vinyl polymerization, ring-opening multibranching polymerization, click chemistry, enzyme-catalyzed polymerization [1,2,3,4]. HBPs have been widely used in biomaterials, nanocomposites, amphiphilic polymers, coatings, crosslinkers, adhesives, membranes, rheology modifiers and functional additives [5,6,7,8], etc. 

Hyperbranched polyurethane acrylates (HBPUA), as a novel functional resin, simultaneously combine the outstanding low viscosity, high functionality and high solubility of HBPs, and excellent photoreactivity, chemical resistance, and adhesion of polyurethane acrylates (PUA). These excellent properties make them quite suitable for the green, environmental and efficient trend of UV-curable formulations, which should possess high molecular weight, low viscosity and high reactivity [9,10]. 

HBPUA are mainly synthesized by modifying different hydroxyl-terminated hyperbranched polymers (HBP–OH), such as hyperbranched polyester [11,12,13,14,15,16,17,18], hyperbranched polyurethane [19], hyperbranched polyether [20], and hyperbranched poly(amine-ester) [21] with various semiadduct urethane monoacrylate of IPDI (isophorone diisocyanate)-HEMA (2-hydroxyethyl methacrylate) [11,12,13], TDI (toluene diisocyanate)-HEA (hydroxy-ethyl acrylate) [14,15,20] or IPDI-HEA [16,17,18,19,21]. Moreover, if HBP–OH partially react with maleic anhydride [22,23,24,25] or succinic anhydride [26,27,28] and neutralize with triethylamine, waterborne hyperbranched polyurethane acrylates can also be produced. HBPUA have been widely applied to UV-curable coatings, adhesives, printing inks and composites to reduce viscosity, accelerate curing reaction, and enhance mechanical strengths and toughness of other materials.

At present, HBPs and HBPUA have been widely utilized as functional additives and modifiers, but it is difficult for them to directly form freestanding films. Due to the nature of compactly packed periphery, high functionality and lack of chain entanglements, their freestanding films are very stiff but brittle [29,30]. To ameliorate the brittleness without sacrificing the unique architecture of HBPs, some strategies have been attempted, such as grafting reactive polymer chains [31,32], modification of end-group with short flexible chains [33,34,35] and copolymerization with their linear analogues [36,37]. However, it is still difficult to produce freestanding UV-cured HBPUA films with excellent flexibility (<3 mm) [11,16]. 

In this study, flexible polyethylene glycol (PEG200) segment is firstly reacted with IPDI and HEA to form flexible semiadduct urethane monoacrylate (IPDI/PEG200/HEA) which then react with hydroxy terminate hyperbranched polyurethane (HBPU–OH), to develop flexible hyperbranched polyurethane acrylates (FHBPUA). The properties of UV-cured freestanding FHBPUA films and photopolymerization kinetics are studied. After that, FHBPUA is applied to epoxy acrylate resin to improve its UV-curing and mechanical performances.

## 2. Experimental Section

### 2.1. Materials

Isophorone diisocyanate (IPDI), polyethylene glycol (PEG, *M*_n_ = 200), hydroxyethyl acrylate (HEA), diethanol amine (DEA), dimethylacetamide (DMA), dibutyltin dilaurate (DBTDL), hydroquinone monomethyl ether (MEHQ), dibutylamine and pyridine were purchases from Aladdin Reagent Co. (Shanghai, China) and used without further purification. Commercial available bisphenol A epoxy acrylate resin (UV1000) kindly provided by Qianyou Chemical Materials Co., Ltd (Zhongshan, China).

### 2.2. Preparation of Hydroxyl Terminated Hyperbranched Polyurethane (HBPU–OH)

**Scheme 1:**
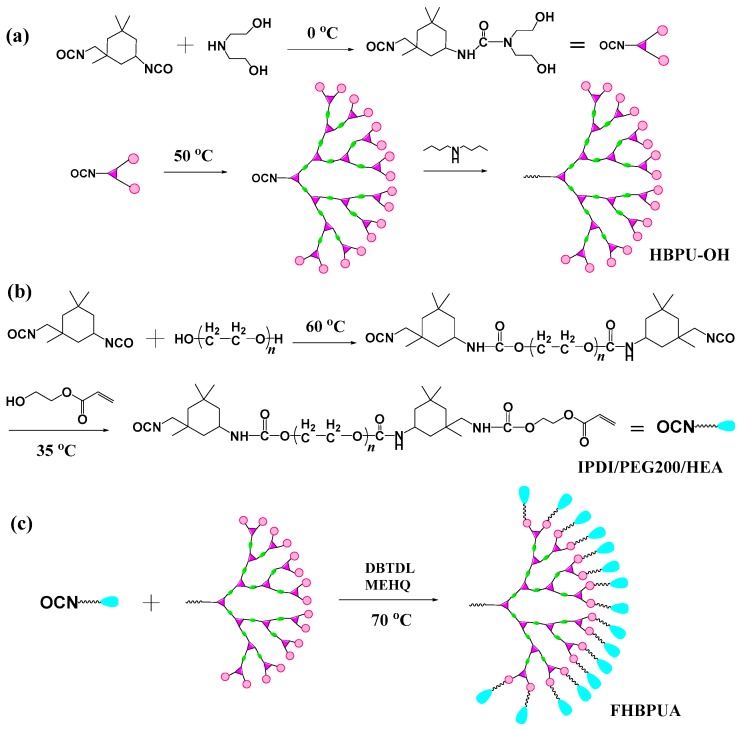
Synthesis routes of FHBPUA. (**a**) preparation of HBPU–OH; (**b**) preparation of IPDI/PEG200/HEA; (**c**) reaction between HBPU–OH and IPDI/PEG200/HEA.

The fourth generation of hydroxyl-terminated hyperbranched polyurethane (HBPU–OH) was synthesized according to Scheme 1a. IPDI (33.33 g, 0.15 mol) and 35 mL DMA were added to a four-necked flask equipped with a mechanical stirrer, nitrogen inlet, thermometer, and dropping funnel. As the mixtures were cool to 0 °C, DEA (15.77 g, 0.15 mol) and 16 mL DMA were added dropwise, and reacted for 2 h. Then, the temperature was slowly heated up to 50 °C. The NCO content was determined every 20 min. Excess dibutylamine was added to terminate reaction as soon as the NCO content was equal to its theoretical value (1.83%~0.86%) calculated from its potential structures [38]. After precipitating with ether and vacuum-drying, HBPU–OH with a yield of ~86% obtained. 

### 2.3. Preparation of Semiadduct Urethane Monoacrylate (IPDI/PEG200/HEA)

The semiadduct urethane monoacrylate (IPDI/PEG200/HEA) was synthesized as Scheme 1b. IPDI (22.22 g, 0.10 mol) and 25 mL DMA were well-mixed, and then PEG200 (10 g, 0.05 mol) and 10 mL DMA was added dropwise. After that, the mixtures were reacted at 60 °C. As the NCO content was close to its theoretical value, the temperature was cool to 35 °C. HEA (7.55 g, 0.065 mol), DBTAL (0.037 wt %), MEHQ (0.1 wt %) and 10 mL DMA were dropped into the mixtures, and reacted at 35 °C for about 2 h. As the NCO content reached its theoretical value, the reaction was terminated and IPDI/PEG200/HEA obtained. 

### 2.4. Preparation of Flexible Hyperbranched Polyurethane Acrylate (FHBPUA)

As shown in Scheme 1c, HBPU–OH was mixed with IPDI/PEG200/HEA, in which the molar ratio of OH in HBPU–OH-4 and NCO in IPDI/PEG200/HEA was 1:1. After adding DBTAL (0.05 wt %) and MEHQ (0.1 wt %), the mixture was reacted at 70 °C until the peak at 2270 cm^−1^ for NCO groups in infrared spectrum completely disappeared. After precipitating with ether, washing with acetone and vacuum drying, FHBPUA with a yield of 89% obtained.

### 2.5. Preparation of UV-Cured Films

The freestanding film of FHBPUA was prepared as following, FHBPUA dissolved into anhydrous ethanol to form 85 wt % solutions, mixed with 4 wt % Irgacure1173 as photoinitiator, and molded by an automatic film coating machine (AFA-II, Shanghai Modern Environment Engineering Technique Co., Ltd., Shanghai, China) with a typical thickness of 30 μm. After drying at 60 °C for 5 min, irradiating under 365 nm UV light with an intensity of 30 mW/cm^2^, UV-cured FHBPUA film obtained. For the UV1000 and FHBPUA composite film, FHBPUA was firstly dissolved in a little of ethyl alcohol, and then mixed with UV1000 and Irgacure1173 (4 wt %). The mixture was exposed to UV irradiation to produce films until ethyl alcohol was completed volatilized under vacuum.

### 2.6. Characterization

Fourier transform infrared spectroscopy (FTIR) measurements in KBr pellets were carried out using a Nicolet Magna 360 spectrometer from 4000 cm^−1^ to 400 cm^−1^, with a resolution of 4 cm^−1^. Nuclear magnetic resonance (NMR) spectra were determined by a AVANCE III HD 400 MHz spectroscopy (Bruker, Fällanden, Switzerland), DMSO-*d_6_* was used as the solvent. The isocyanate content (NCO %) was determined by chemical titration according to ASTM D5155-01 standard. Pencil hardness of the UV-cured film was estimated in accordance with ASTM D3363. Adhesion force was measured according to ISO2409-1992 standard. The flexibility of the UV-cured films was tested according to ASTM D4338-97 standard. The gloss under 60° was measured by means of ASTM D523 standard. The molecular weight and polydispersity of FHBPUA were measured by gel permeation chromatography (GPC, Waters 1515, Milford, MA, USA) with polystyrene as calibration standard and tetrahydrofuran (THF) as eluent. Tensile tests were measured at room temperature with a universal tester (SANS CMT 6000, Shenzhen, China). The dumb-bell shaped specimens were prepared and tested with a crosshead speed of 25 mm/min according to ASTM D412. The glass transition temperature was determined by differential scanning calorimetry (DSC) with TA Instruments Q10 (New Castle, DE, USA) under N_2_ atmosphere and a heating rate of 10 °C/min from 0 to 180 °C. Thermal gravimetric analysis (TGA) was carried out on STA 499C (NETZSCH, Selb, Germany) in N_2_ atmosphere from 40 to 800 °C by 10 °C/min. The UV-curing kinetics was studied by Real-Time FTIR (Nicolet 6700, ThermoFisher Scientific, Waltham, MA, USA) with a horizontal sample holder. A high-pressure mercury lamp (MUA-165, MEJIRO GENOSSEN, Tokyo, Japan) was directed to the samples. The UV source was in the range of 250~365 nm, and the distance between the UV light and sample was controlled to be 10 ± 1 cm to guarantee the UV intensity. The double bond conversion (*DC*) was calculated from the decay of absorption peak at 810 cm^−1^ belonged to acrylate double bond, and the characteristic absorption peak of carbonyl group (C=O) at 1720 cm^−1^ was used as the internal standard. The *DC* was calculated with Equation (1) [39].

*DC*/% = [(*A*_810_/*A*_1720_)*_t_* − (*A*_810_/*A*_1720_)_0_]/(*A*_810_/*A*_1720_)_0_ × 100 (1)

where (*A*_810_/*A*_1720_)_0_ and (*A*_810_/*A*_1720_)*_t_* were the relative peak area ratio of acrylate double bond to carbonyl group before and after UV irradiation curing at different time (*t*), respectively. The rate of photopolymerization was estimated by the differential of double bond conversion versus irradiation time [40].

## 3. Result and Discussion 

### 3.1. Characterization of FHBPUA 

**Figure 1:**
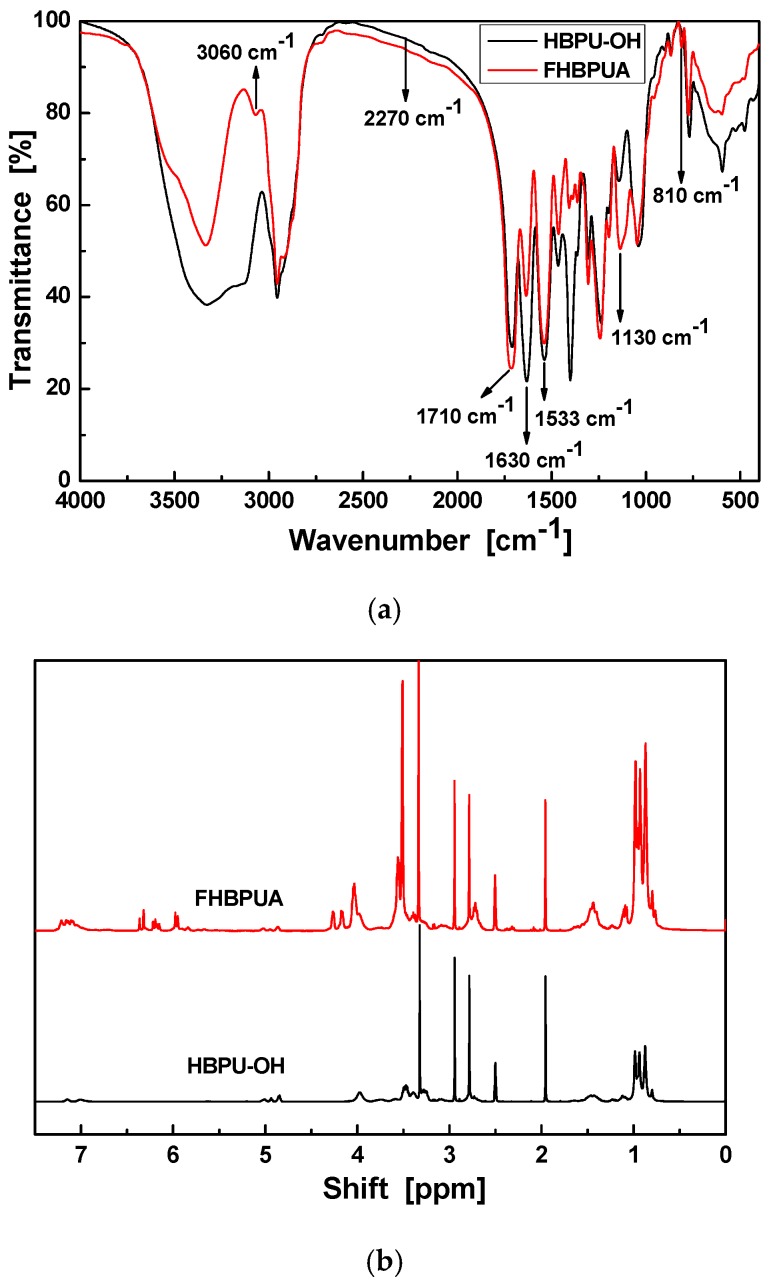

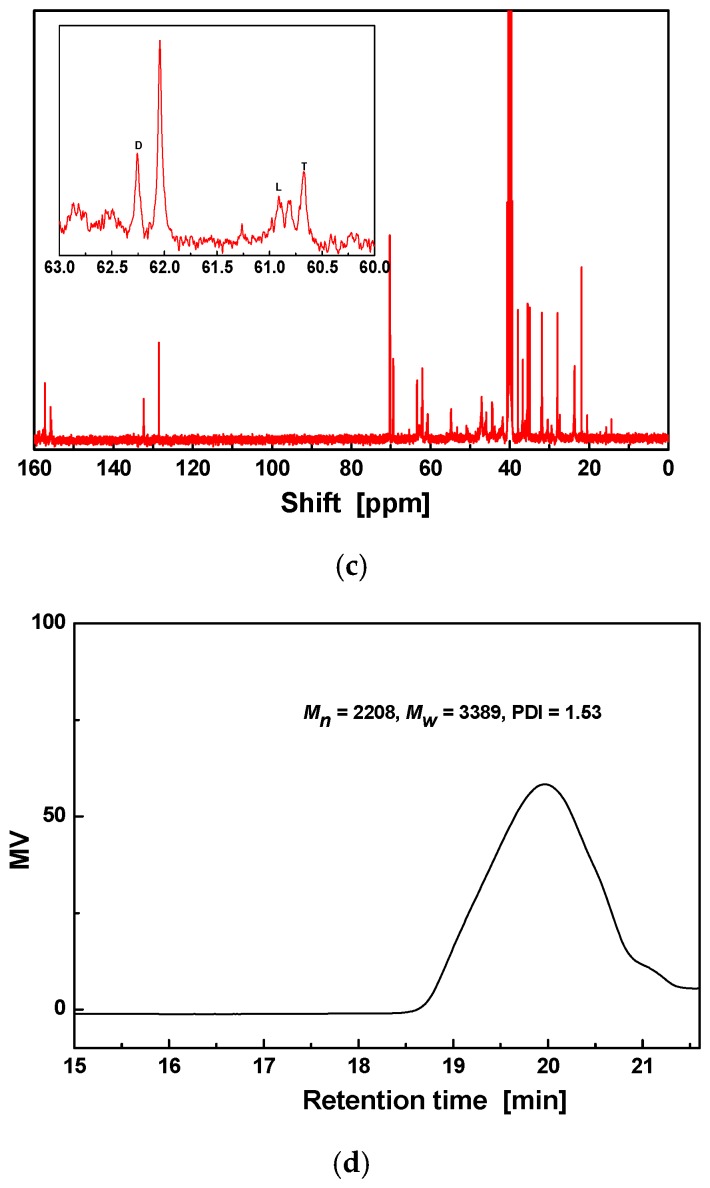
Characterization of FHBPUA. (**a**) Fourier transform infrared spectroscopy; (**b**) ^1^H nuclear magnetic resonance; (**c**) ^13^C nuclear magnetic resonance and (**d**) Gel permeation chromatography.

As FTIR spectra of HBPU–OH and FHBPUA in Figure 1a, the broad peak at 3000~3700 cm^−1^ of HBPU–OH is mainly assigned to terminal hydroxyls. As HBPU–OH reacts with IPDI/PEG200/HEA, the strong peak at 3100~3700 cm^−1^ of FHBPUA should be ascribed to the stretching vibration of –NH, and the bending vibration peak of –NH occurs at 1533 cm^−1^. Compared with HBPU–OH, it is clear that two new characteristic peaks appeared at 3060 cm^−1^ and 810 cm^−1^ in the curve of FHBPUA, which are attributed to the stretching vibration and bending vibration of –CH=CH_2_, respectively. The absorption peak of C–O–C at 1130 cm^−1^ [41] for FHBPUA is also greatly enhanced owing to the introduction of PEG200. The peak at 1630 cm^−1^ is assigned to C=O of –NHCON– resulted from the reaction of –NCO and –NH, but the peak at 1710 cm^−1^ is ascribed to C=O in –NHCOO– owing to the reaction between –NCO and –OH [42]. It means that the IPDI/PEG200/HEA has successfully reacted with HBPU–OH. Additionally, the absence of the characteristic peak of –NCO at 2270 cm^−1^ implies that the NCO groups have been completely reacted.

Compared with HBPU–OH, three characteristic peaks at 5.94~6.38 ppm, 4.14~4.29 ppm and 3.45~3.62 ppm are revealed in ^1^H NMR spectra of FHBPUA in Figure 1b, which can be assigned to –CH=CH_2_ of HEA, –CH_2_–CH_2_– of HEA and –CH_2_–CH_2_– of PEG, respectively [19]. It indicates the existence of HEA and PEG. The peak at 7.04~7.27 ppm is ascribed to N–H of –NHCOO– and –NHCON–. The peaks at 0.65~2.95 ppm are mainly ascribed to –CH–, –CH_2_– and –CH_3_ groups [19]. In Figure 1c, the resonance at 155.41~157.57 ppm, 132.57 ppm, 69.08~70.52 ppm, and 63.19 ppm can be ascribed to C=O of –NHCON– and –NHCOO–, –CH=CH_2_ of HEA, –CH_2_–CH_2_– of PEG, and –CH_2_–CH_2_– of HEA, respectively. The peaks at 62.26 ppm, 60.91 ppm, and 60.67 ppm can be severally ascribed to the dendritic unit (D), linear unit (L) and terminal unit (T) in FHBPUA [42,43]. According to Fréchet Equation (2) [44], the degree of branch (*DB*) of FHBPUA turns out to be 0.82, which is close to its analogues [38].

*DB* = (D + T)/(D + T + L) (2)

The molecular weights and polydispersity of FHBPUA are analyzed by GPC in THF, using linear polystyrene standard. It is well known that hyperbranched polymers have much lower hydrodynamic volumes than linear polymers with the same molecular weight, which lead to the molecular weight obtained by GPC is much lower than the theoretical value [16,45]. From Figure 1d, the weight-average molecular weight (*M*_W_) is 3389 g/mol, which is much lower than its theoretical value (3999~13,494 g/mol). However, the polydispersity of hyperbranched polymers from GPC is reasonably accurate and can be used. The polydispersity of FHBPUA is 1.53.

### 3.2. Thermal Properties 

**Figure 2:**
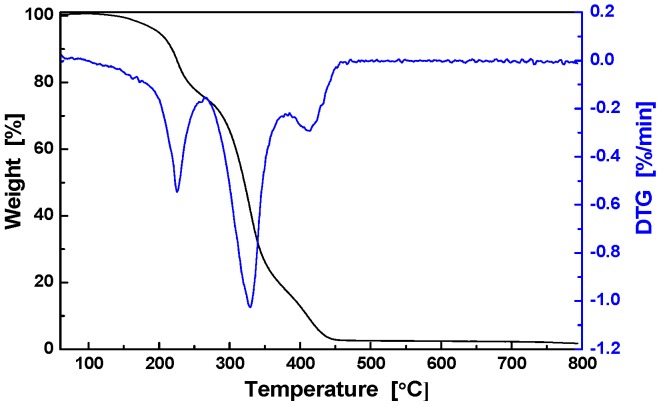
Thermogravimetric analysis curves of FHBPUA.

The thermal stability of FHBPUA is characterized by thermogravimetric analysis (TGA) in Figure 2, which shows three thermal degradation behaviors and the second step is the most significant [18,46]. In the first step, the maximum decomposition rate occurs at 225 °C, owing to the decomposition of urethane bonds in hard segments. The second stage arranges from 260 to 380 °C and the maximum decomposition rate is at 330 °C, which result from the decomposition of polyether soft segments. The maximum decomposition rate in the last step is at 415 °C, due to the decomposition of imide structures [18,46]. Additionally, the glass transition temperature of FHBPUA determined by DSC is around 65 °C, which is much lower than that of HBPUs (107~132 °C) without flexible segments [38].

### 3.3. Photopolymerization Kinetics

**Figure 3:**
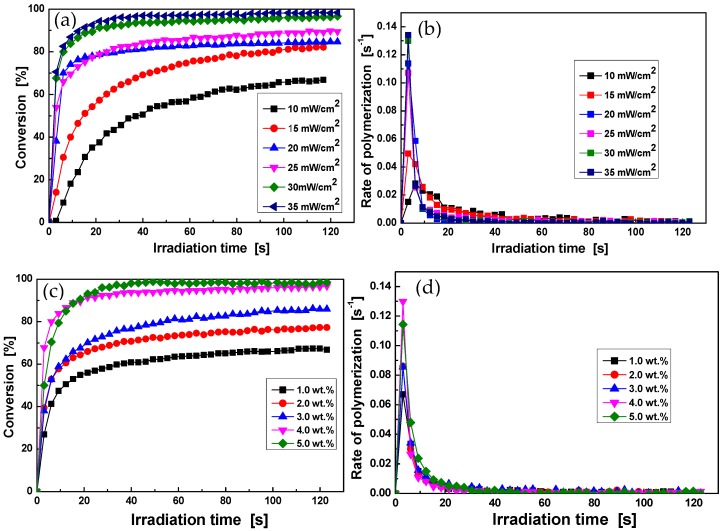

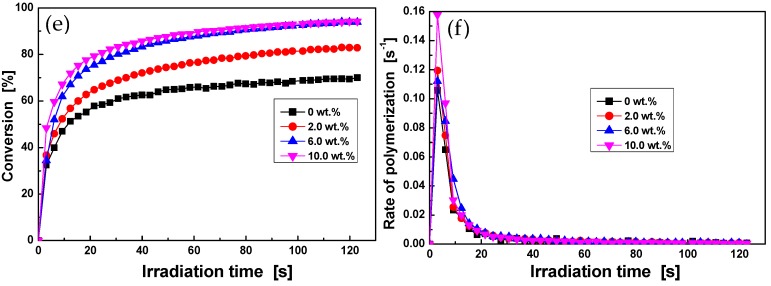
Effect of light intensity and dosage of photoinitiator on photopolymerization kinetics of FHBPUA and UV1000 resin; (**a**) double bond conversion and (**b**) rate of photopolymerization as a function of irradiation time with 4.0 wt % Irgacure 1173 under different light intensities; (**c**) double bond conversion and (**d**) rate of photopolymerization as a function of irradiation time under 30 mW/cm^2^ with various dosage of Irgacure 1173; (**e**) double bond conversion and (**f**) rate of photopolymerization of UV1000 resin containing different dosage of FHBPUA as a function of irradiation time with 4.0 wt % Irgacure 1173 and 30 mW/cm^2^ light intensity.

The properties of the UV-cured films rely not only on the compositions but also on the photopolymerization kinetics. The most important parameters for photopolymerization kinetics are the rate of photopolymerization and degree of double bond conversion as a function of irradiation time. The conversion and rate of photopolymerization versus irradiation time curves of FHBPUA under different light intensities are shown in Figure 3a,b. It can be seen that both conversion and rate of photopolymerization increase with the increase of light intensity. This can be attributed to the higher active radical species concentration and a temporary excess of free volume [47,48]. From the energy and cost saving point, 30 mW/cm^2^ should be a proper light intensity. 

The dosage of photoinitiator is another key factor in photopolymerization kinetics. Figure 3c,d demonstrate both photopolymerization rate and conversion enhance with the increase of photoinitiator dosage from 1.0 wt % to 4.0 wt %, whilst the further increase to 5.0 wt % dose not lead to the significant improvement of conversion and photopolymerization rate. This is because the free radicals easily conjugate and terminate the propagation reaction, especially when a high dosage of photoinitiator can generate excessive free radicals. Additionally, excessive initiator molecules in the surface layer may absorb a great deal of light energy, which lead to the lower light energy transmitted to a deep layer, so the conversion and photopolymerization rate in the deep layer are reduced [40,48]. Consequently, the conversion and photopolymerization rate are not notably increased as the dosage of photoinitiator increases to a certain degree. 

The influence of FHBPUA on photopolymerization kinetics of UV1000 resin is shown in Figure 3e,f. The double bond conversion is increased from 70 to 94% as the dosage of FHBPUA increases to 6%, but it maintains at ~94% even if FHBPUA furtherly increases to 10%. The rate of photopolymerization is gradually enhanced with FHBPUA. The vast and high photoreactive acrylate double bonds in FHBPUA facilitate the photopolymerization reaction [23], and the soft chains in FHBPUA can further enhance the autoacceleration effect of free radical photopolymerization [49]. Therefore, a certain amount of FHBPUA can improve the conversion and rate of photopolymerization of UV1000 resin.

### 3.4. Properties of UV-Cured Films

The properties of UV-cured freestanding FHBPUA film and epoxy acrylate (UV1000) composite film are listed in Table 1. The FHBPUA film can be fully UV-cured within 3 s, and its flexibility up to 1 mm is remarkably superior to the reported 3~8 mm [11,16]. The curing time of UV1000 composite film can be substantially reduced from 6 s to 3 s with the addition of FHBPUA. This is due to a large amount of photoreactive acrylate double bonds on the surface of FHBPUA, which effectively promotes the photopolymerization reaction [23]. However, as the reaction rate reaches its maximum, the curing time cannot be shortened even if more FHBPUA is added [15]. The flexibility of UV1000 film is tremendously enhanced from 10 mm to 1 mm as the FHBPUA amount increases to 10 wt %, resulting from the flexible chains in FHBPUA [50]. The pencil hardness of UV1000 is slightly decreased from 3H to 2H, due to the introduction of soft segment in FHBPUA. With the increase of FHBPUA content to 10 wt %, the adhesive force is also enhanced from five grades to three grades, resulting from the stronger intermolecular interaction between the polar groups in FHBPUA and metal plates [46]. Additionally, the addition of FHBPUA into UV1000 film has little influence on its glossiness, which implies they have good compatibility [51] and ensures the industrial applications of FHBPUA.

**Figure 4:**
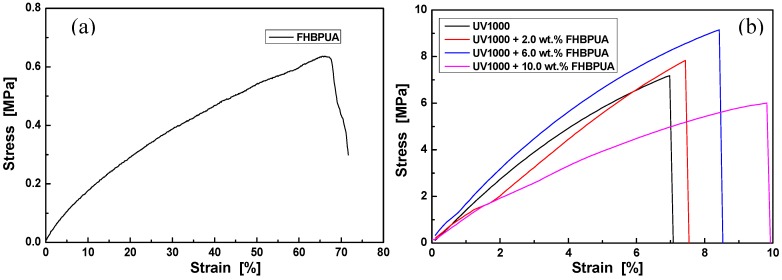
Tensile stress-strain curves of UV-cured film (**a**) FHBPUA and (**b**) UV1000 resin.

The stress-strain curves of UV-cured films are shown in Figure 4. The tensile strength and elongation at break of FHBPUA film in Figure 4a are 0.63 MPa and 67%. In Figure 4b, the tensile strength and elongation at break of pure UV1000 film are 7.15 MPa and 6.98%, and its mechanical strengths can be improved by moderate amounts of FHBPUA. After adding 6.0 wt % FHBPUA, the tensile strength and elongation at break of UV1000 composite film reach 9.12 MPa and 8.41%. However, the tensile strength drops to 6.00 MPa, while the elongation at break further increases to 9.77% with increasing FHBPUA to 10.0 wt %. A certain amount of FHBPUA added can increase crosslink networks after UV irradiation owing to its superior photosensitivity, thus the tensile strength and elongation at break are improved [15]. Nevertheless, the tensile strength can also be deteriorated by excessive FHBPUA because a mass of PEG200 chains soften the films according to the decrease of pencil hardness from 3H to H in Table 1.

## 4. Conclusions

The mechanical brittleness of UV-curable hyperbranched polyurethane acrylates is effectively addressed and produces freestanding flexible hyperbranched polyurethane acrylates (FHBPUA) films via modifying hydroxyl-terminated hyperbranched polyurethane (HBPU–OH) with flexible semiadduct urethane monoacrylate. The degree of branching of FHBPUA obtained is 0.82, and its *T_g_* is around 65 °C. The FHBPUA film can be UV-cured within 3 s, and its flexibility reaches 1 mm. With the increase of FHBPUA in epoxy acrylate (UV1000) film, the UV-curing time of UV1000 film is decreased to 3 s. The flexibility of UV1000 film is greatly improved to 1 mm, and the adhesion force is also increased to three grades. Moreover, the addition of FHBPUA does not distinctly affect the glossiness of UV1000 film, implying they have good compatibility. The tensile strength and elongation at break of UV1000 film are also reinforced by moderate amounts of FHBPUA. This novel FHBPUA is expected to directly develop flexible films, as well as functional additives to perfect other UV-curable formulas.

## Figures and Tables

**Table 1:** Properties of UV-cured films of FHBPUA and UV1000 resin.

Samples	FHBPUA	Curing Time/s	Pencil Hardness	Flexibility/mm	Adhesive Force/Grade	Glossiness/Gu
FHBPUA	100%	3	HB	1	3	92.1
UV1000	0%	6	3H	10	5	98.3
	2%	3	3H	10	5	97.7
	4%	3	3H	5	4	97.6
	6%	3	2H	3	4	97.8
	8%	3	2H	2	4	97.1
	10%	3	H	1	3	97.4
